# Air and Exercise

**Published:** 1858-03

**Authors:** 


### AIR AND EXERCISE.

ANY one may observe that exercise increases the rapidity of our breathing, hence the faster we breathe the more air we consume, and as the blood is purified by the air, the argument will run thus:

The greater the exercise the greater is the rapidity of breathing; the greater is the amount of air consumed, the more perfectly is the blood purified, and the more robust health will we enjoy. *Inference*—EXERCISE PROMOTES HEALTH! *Quod erat demonstrandum.*

In this connection, it may be useful to state, that it is estimated that, under ordinary circumstances as to sedentary persons, a young man should walk six miles a day, a young woman four. When at rest, we breathe a gallon and a half of air, about 500 cubic inches, that is, consume the oxygen which it contains.

In walking at the rate of one mile an hour, we consume in a minute—of air . . . . . . . . . . 800 cubic inches.

| | | | |
|---|---|---|---|
| " | two miles . | 1,000 | " |
| " | three miles . | 1,600 | " |
| " | four miles . | 2,300 | " |
| " | six miles . | 3,000 | " |
| Cantering on horseback . . . . | | 1,500 | " |
| Trotting . . . . . . . . . . . | | 1,750 | " |

When persons are so strong that they are feeling better while walking fast than walking at a moderate pace, and stopping before great fatigue comes on, it is always promotive of health—provided that the pedestrian cools off very slowly, out of draughts in Summer-time, and by a fire in cold weather. If, while a person is walking out, every step is a drag, then every step is an injury.

If a patient is too weak to walk without fatigue, approaching to exhaustion, then horseback exercise, or riding in an open carriage, is better.

Spasmodic exercise, that which is short and violent, is contrary to nature, it is seldom unattended with injurious consequences, and as to invalids, causes death sometimes.

